# Calcium and autophagy connect photorespiratory oxidative signals with salicylic acid–mediated defense

**DOI:** 10.1093/plphys/kiag741

**Published:** 2026-10-07

**Authors:** Yunti Ge, Bo Xu

**Affiliations:** ARC Centre of Excellence in Plants for Space, and School of Agriculture, Food and Wine, Waite Research Institute, Adelaide University, Glen Osmond, SA 5064, Australia; ARC Centre of Excellence in Plants for Space, and School of Agriculture, Food and Wine, Waite Research Institute, Adelaide University, Glen Osmond, SA 5064, Australia; Assistant Features Editor, Plant Physiology, American Society of Plant Biologists

In plants, photorespiration occurs in photosynthetically active tissues—such as mesophyll cells and guard cells—and is initiated in chloroplasts when Rubisco (ribulose-1,5-bisphosphate carboxylase/oxygenase) reacts with oxygen (O_2_), producing a 3-carbon 3-PGA (3-phosphoglycerate) and a 2-carbon 2-PG (2-phosphoglycolate). 2-PG is subsequently converted to glycolate and transported to peroxisomes, where glycolate-oxidase (GOX) oxidizes it to glyoxylate, producing hydrogen peroxide (H_2_O_2_) (Foyer et al 2009) (Fig. 1). H_2_O_2_, a relatively stable form of reactive oxygen species (ROS), acts as an intracellular signaling molecule but can cause oxidative damage when excessively produced (Waszczak et al 2018). Under normal conditions, H_2_O_2_ from photorespiration is efficiently removed by peroxisomal catalases, restricting oxidative stress (Foyer et al 2009) (Fig. 1).

**Figure 1:**
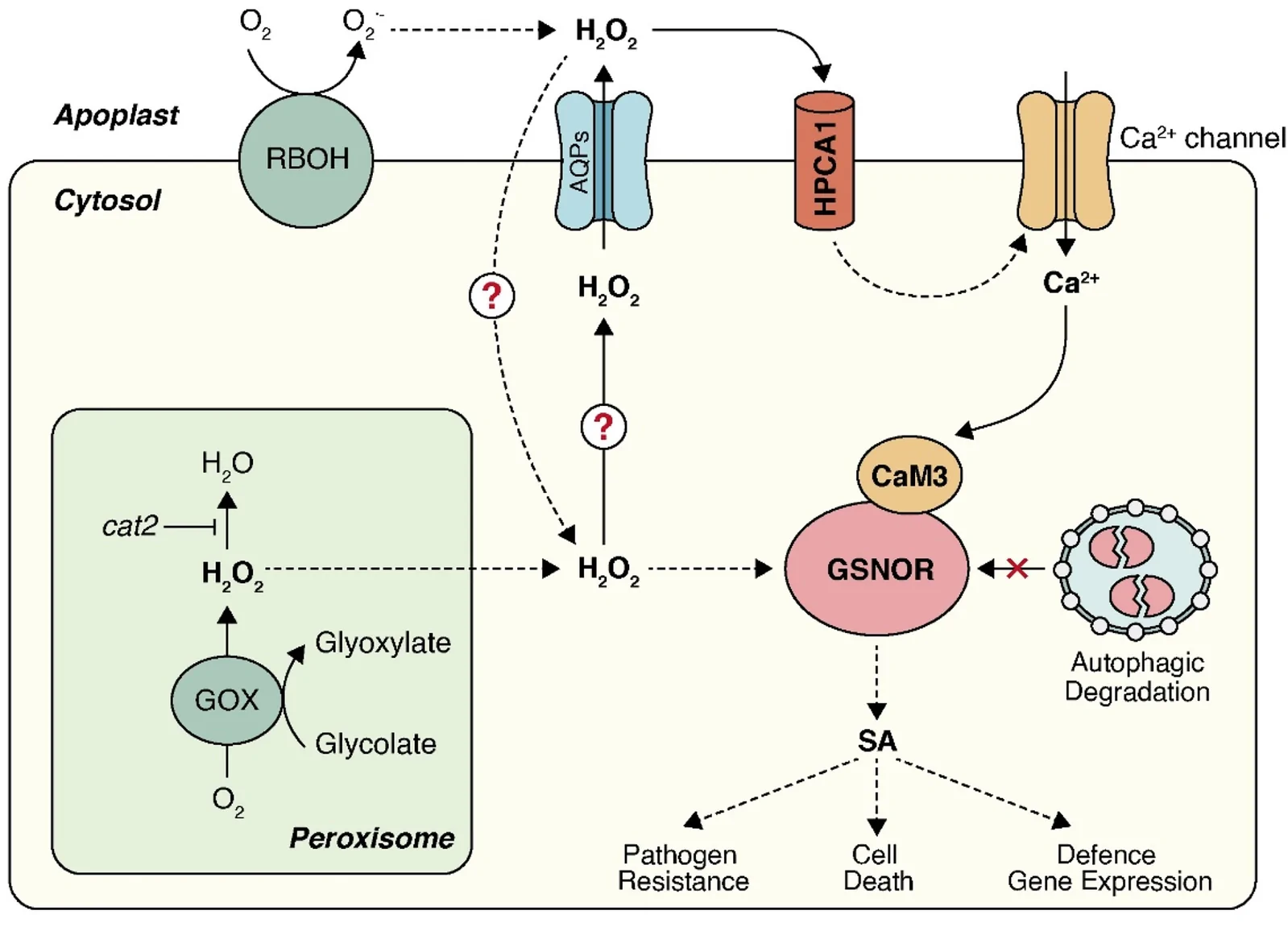
An updated model of HPCA1-CaM3-GSNOR signaling cascade linking photorespiratory H_2_O_2_ and SA responses in *cat2*. In Arabidopsis, photorespiration produces H_2_O_2_ in peroxisomes when GOX converts glycolate to glyoxylate; this H_2_O_2_ is normally efficiently scavenged by CAT2. Loss of *CAT2* impairs photorespiratory H_2_O_2_ scavenging (Queval et al 2007) and promotes HPCA1-dependent Ca^2+^ signaling at the plasma membrane. Ca^2+^-activated CaM3 interacts with GSNOR1 and restricts its autophagic degradation, thereby maintaining GSNOR1 protein abundance and activity. Enhanced GSNOR1 activity promotes SA biosynthesis and signaling, cell death, and bacterial pathogen resistance in *cat2* (Luo et al 2026). Here, how peroxisomal H_2_O_2_ is communicated to the apoplastic H_2_O_2_ to activate HPCA1 remains unresolved. RBOHF-dependent apoplastic ROS production has been proposed to contribute to this communication, as indicated by a question mark (Luo et al 2026). Another complementary hypothetical route involves plasma-membrane aquaporins (AQPs), which can facilitate H_2_O_2_ efflux from the cytosol to the apoplast when intracellular H_2_O_2_ is excessive (Zhang et al 2023), which remains to be tested in a *cat2* mutant, as indicated by a question mark. Dashed lines represent proposed multistep pathways, whereas solid lines denote likely direct effects. Adapted from Luo et al (2026) and extended with a proposed AQP-mediated H_2_O_2_ efflux based on Zhang et al (2023).

In *Arabidopsis thaliana*, CATALASE 2 (CAT2) is the major catalase in leaves, and *cat2* mutants only retain ∼10% to 15% of wild-type leaf catalase activity (Queval et al 2007). Under ambient CO_2_ and long-day growth conditions, *cat2* displays pronounced leaf lesions and induces the expression of ROS-responsive marker genes (eg, *OXI1* [*oxidative signal-inducible 1*]), suggesting that impaired photorespiratory H_2_O_2_ scavenging causes strong redox perturbation (Queval et al 2007). Loss of *CAT2* also constitutively activates the salicylic acid (SA) pathway, resulting in increased SA biosynthesis and induction of SA-marker genes—*PR1* (*pathogen-related 1*), *PR2*, and *ICS1* (*isochorismate synthase 1*). These constitutive SA responses have been associated with enhanced S-nitrosoglutathione reductase 1 (GSNOR1) activity in *cat2* (Zhang et al 2020) (Fig. 1). GSNOR metabolizes S-nitrosoglutathione (GSNO) (a reservoir of nitric oxide [NO]) to oxidized glutathione and ammonia (NH_3_), thereby regulating cellular S-nitrosothiol homeostasis; its activity is sensitive to the cellular redox state (eg, H_2_O_2_) (Li et al 2021). Disruption of *GSNOR1* partially suppressed *cat2* phenotypes, greatly abolishing leaf lesion, SA accumulation, and SA-marker gene induction. These findings place GSNOR1 downstream of photorespiratory H_2_O_2_ signaling (Zhang et al 2020). Moreover, GOX activity was similarly reduced in both *cat2* and *cat2 gsnor1* double mutants, indicating that suppression of SA signaling in *cat2 gsnor1* was not caused by a further reduced H_2_O_2_-generating capacity (Zhang et al 2020). By contrast, loss of *GOX1* in *cat2* partially attenuated lesion formation and SA-signaling activation, confirming that GOX1-mediated H_2_O_2_ formation also contributes to the *cat2* phenotype (Yang et al 2025). Together, these findings demonstrate GSNOR1-modulated protein S-nitrosation status as a key mechanism linking photorespiratory H_2_O_2_ to SA signaling (Zhang et al 2020; Yang et al 2025), although how impaired photorespiratory H_2_O_2_ scavenging enhances GSNOR1 activity remains unknown.

In a recent study published in *Plant Physiology*, Luo et al (2026) addressed this gap by showing that H_2_O_2_-induced calcium (Ca^2+^) signaling links photorespiratory H_2_O_2_ to GSNOR1-dependent SA signaling and disease resistance through regulation of GSNOR1 stability. High-throughput yeast two-hybrid sequencing identified several hundred putative GSNOR1-interacting proteins, among which the Ca^2+^ sensor calmodulin 3 (CaM3) showed the highest read count and was selected for further analysis. Interestingly, the CaM3-GSNOR1 interaction was enhanced by pretreatment with either H_2_O_2_ or catalase inhibitors, supporting that oxidative stress promotes the association between CaM3 and GSNOR1 (Luo et al 2026). The *cat2 cam3* double mutants had reduced GSNOR1 protein abundance and activity, and *cam3* partially restored *cat2* growth. c*at2 cam3* mutants also accumulated less SA; showed weaker induction of *PR1*, *PR2*, and *ICS1*; and reduced resistance to bacterial pathogen *Pseudomonas syringae* (Luo et al 2026). These findings identify CaM3 as a positive regulator of GSNOR1-dependent SA signaling (Fig. 1).

The involvement of CaM3 implicates that a Ca^2+^ signaling pathway may operate between H_2_O_2_ and CaM3-GSNOR1 module (Luo et al 2026). HPCA1 (hydrogen peroxide induced Ca^2+^ increases 1), a plasma-membrane leucine-rich repeat receptor kinase, senses apoplastic H_2_O_2_ and subsequently facilitates Ca^2+^ influx (Wu et al 2020) (Fig. 1). To determine the contribution of HPCA1 to photorespiratory H_2_O_2_-linked SA signaling, Luo et al (2026) generated *cat2 hpca1* double mutants, which largely phenocopied *cat2 cam3* double mutants. Overexpression of *CaM3* in *cat2* (*cat2 CAM3-OE*) further enhanced GSNOR1 abundance and activity, whereas these effects were attenuated by loss of *HPCA1* in *cat2 hpca1 CAM3-OE*. Together with the established role of HPCA1 as an apoplastic H_2_O_2_ sensor, these genetic data place CaM3 downstream of HPCA1, suggesting that HPCA1-mediated H_2_O_2_ perception is coupled with intracellular Ca^2+^-dependent CaM3 signaling (Wu et al 2020; Luo et al 2026) (Fig. 1).

A previous study showed that inhibition of autophagy increases GSNOR1 abundance through stabilizing the GSNOR1 protein (Li et al 2021) (Fig. 1). Consistently, Luo et al (2026) found that treatment with autophagy inhibitors restored GSNOR1 protein levels in *cat2 cam3* and *cat2 hpca1* to that in *cat2*. Similarly, introduction of the *autophagy-related 18a* (*atg18a*) into *cat2 cam3* and *cat2 hpca1*, generating *cat2 cam3 atg18a* and *cat2 hpca1 atg18a* triple mutants, reversed GSNOR1 protein abundance and activity, SA signaling, and *P. syringae* resistance toward *cat2* levels. As such, autophagic degradation mediates the effects of H_2_O_2_-triggered Ca^2+^ signaling on GSNOR1 protein stability (Fig. 1).

Taken together, this research indicates that HPCA1/CaM3 signaling sustains GSNOR1 abundance and activity by restricting autophagic degradation, providing a mechanistic link between H_2_O_2_-triggered Ca^2+^ signaling and downstream SA signaling (Luo et al 2026). Nevertheless, how impaired scavenging of photorespiratory H_2_O_2_ in peroxisomes is communicated with the apoplastic H_2_O_2_ and perceived by HPCA1 remains unresolved. Luo et al (2026) proposed that RBOHF (respiratory burst oxidase homolog F)-dependent apoplastic ROS production may contribute to this process. A complementary mechanism was suggested by Zhang et al (2023), who identified that *AT1* (*alkaline tolerance 1*), encoding an atypical G protein γ subunit. AT1 regulates the phosphorylation of plasma-membrane PIP2 aquaporins and modulates H_2_O_2_ movement between the cytosol and apoplast. Enhanced PIP2 phosphorylation promoted H_2_O_2_ efflux and apoplastic ROS accumulation (Zhang et al 2023). This raises the possibility that excessive intracellular H_2_O_2_ generated by impaired peroxisomal photorespiratory H_2_O_2_ scavenging may be similarly exported through aquaporins to apoplast for activating HPCA1 (Zhang et al 2023; Luo et al 2026); however, this remains to be experimentally tested (Fig. 1). Notably, guard cell–specific manipulation of photorespiration was recently shown to alter guard cell H_2_O_2_ accumulation and stomatal behavior (Sun et al 2026). This raises another question of whether a similar HPCA1-CaM3-GSNOR1 signaling circuit could be reciprocal in guard cells to couple photorespiratory redox status with stomatal regulation.

## Data Availability

No new data was generated for this News and Views.
